# Physiological Effects of Natural and Artificial Aging of Desert Short-Lived Forage Species and Restoration by Gibberellic Acid Priming

**DOI:** 10.3390/plants15071008

**Published:** 2026-03-25

**Authors:** Jing Zhao, Yi Ding, Sumera Anwar, Xuheng Zhao, Min Zhou, Zhihua Sun, Hongsu He

**Affiliations:** 1 College of Animal Science and Technology, Shihezi University, Shihezi 832003, China; 2 Department of Botany, Government College Women University Faisalabad, Faisalabad 38000, Pakistan

**Keywords:** desert ephemerals, germination indices, GA_3_, lipid peroxidation, antioxidant enzymes, abscisic acid

## Abstract

Seed aging is a major constraint for plant establishment in arid and semi-arid ecosystems, where poor seed vigor directly limits species persistence and restoration success. Desert species are particularly vulnerable to storage- and stress-induced deterioration, yet practical strategies to recover germination capacity in aged seeds remain limited. This study aimed to quantify aging-induced losses in germination performance and to evaluate whether exogenous gibberellic acid (GA_3_) can partially restore seed vigor through physiological, biochemical, and hormonal regulation. Fresh seeds (FS), naturally aged (NA), and artificially aged (AA) seeds of four desert species (*Salsola affinis* C.A.Mey., *Trigonella arcuata* C.A.Mey., *Ceratocarpus arenarius* L., and *Alyssum desertorum* Stapf) were exposed to graded GA_3_ concentrations (0–500 mg L^−1^). Germination indices (GP, GR, GI, VI), antioxidant enzymes (SOD, POD, CAT), lipid peroxidation (MDA), phytohormones (IAA, ABA, cytokinins), and multivariate trait relationships were assessed. Without GA_3_, NA reduced germination potential by 22.8–33.6%, while AA caused more severe losses of 42.4–67.8%, depending on species. Germination rate declined by 15.7–32.5% under NA and 36.4–65.2% under AA. GA_3_ application improved all germination indices up to 200 mg L^−1^ (GA200), which increased GP by 22.8–32.0% and vitality index by 17.0–28.5% compared with GA_0_, whereas GA500 showed diminishing returns. Aging suppressed antioxidant enzymes by 15–20% (NA) and 30–45% (AA) and increased MDA by up to 50%, while GA200 enhanced SOD, POD, and CAT and reduced MDA by 8–18%. Aging also reduced IAA and cytokinins (~28–50%) and increased ABA (27.7–77.4%), with GA200 partially restoring hormonal balance. In conclusion, GA_3_ at an optimal dose (200 mg L^−1^) partially reverses aging-induced physiological and hormonal constraints, improving germination and vigor, although recovery remains limited under advanced deterioration.

## 1. Introduction

High-quality seeds form the foundation for sustainable agriculture and animal husbandry. They are also the key biological material for crop improvement and the preservation of valuable germplasm resources [1]. After maturity, seeds often undergo long-term storage, during which their viability gradually decreases, a process known as seed aging [2]. Seed aging is a natural and inevitable process that occurs during storage and in soil seed banks, leading to a gradual decline in germination capacity and seed vigor over time [3]. To experimentally study this long-term process within a practical timeframe, artificial aging is commonly employed by exposing seeds to elevated temperature and relative humidity, thereby accelerating metabolic activity and deterioration. Artificial aging is widely used because it reproduces key physiological and biochemical features of natural aging, while allowing for controlled and reproducible assessment of aging responses and seed vigor loss [4,5].

Seed germination relies heavily on enzymatic activity, as enzymes catalyze essential physiological and biochemical reactions. Changes in their activity directly reflect alterations in seed vigor during the aging process [6]. During seed aging, seeds experience an increase in the production of reactive oxygen species (ROS), including superoxide radicals, hydrogen peroxide, and hydroxyl radicals, which disrupt cellular redox homeostasis [7,8]. To counteract ROS toxicity, seeds rely on antioxidant defense systems, particularly enzymatic antioxidants such as superoxide dismutase, peroxidase, and catalase [9]. However, prolonged or accelerated aging weakens these defense mechanisms, leading to reduced antioxidant capacity and increased susceptibility to oxidative injury, ultimately impairing germination and seed vigor [5].

Seed germination is tightly regulated by endogenous hormonal balance, which is profoundly altered during seed aging. Both natural and artificial aging are associated with a decline in growth-promoting hormones, particularly gibberellins and auxin, alongside a concomitant accumulation of abscisic acid (ABA), resulting in a hormonal environment unfavorable for germination [10,11]. Artificial aging accelerates this imbalance by rapidly suppressing GA biosynthesis and enhancing ABA dominance, thereby intensifying germination inhibition. Previous studies have shown that aging-induced shifts toward reduced GA and elevated ABA are closely linked to loss of seed vigor and delayed or failed germination [5,12].

Desert ecosystems represent some of the most ecologically fragile regions of the world, facing major challenges in vegetation restoration and environmental stability. Among their key species, short-lived desert forage plants serve as ecological pioneers due to their short life cycles and high tolerance to environmental extremes [13,14]. These species play essential roles in wind erosion control, soil improvement, and the supply of early-season forage for grazing livestock. Despite their ecological importance, the seeds of these plants are highly vulnerable to stress factors such as high temperature, drought, and intense solar radiation, leading to natural seed aging in the field [13]. Furthermore, improper storage during propagation can induce artificial aging, resulting in reduced germination rate, weakened vigor, disrupted antioxidant enzyme activity, and hormonal imbalance. Together, these effects limit natural regeneration and hinder the restoration and utilization of desert forage resources [15].

Short-lived desert plants, distributed across the temperate deserts of the southern mountain zone of Shihezi, Xinjiang Province, complete their life cycle rapidly following early spring rainfall. These plants are vital for maintaining ecosystem productivity and biodiversity in desert regions where other vegetation remains dormant [16,17]. *Salsola affinis* C.A.Mey., *Trigonella arcuata* C.A.Mey., *Ceratocarpus arenarius* L., and *Alyssum desertorum* Stapf are desert halophyte plants. They were selected in the present study because of the contrasting life history traits and seed characteristics. *S. affinis* is a halophytic shrub, and its dry, utricle-type seeds exhibit moderate dormancy and gradual viability loss, representing typical salt-desert annuals [17,18]. Many *Salsola* species, including *S. affinis*, demonstrate seed heterochrony in which seeds from the same plant germinate at different times rather than synchronously [19]. *T. arcuata* is a leguminous annual plant with a short life cycle. It produces hard seeds that possess physical dormancy (seed coat–imposed) and are naturally long-lived and resistant to environmental changes [16,20,21].

*C. arenarius* is an annual xerophytic herb adapted to arid and nutrient-poor environments. In China, its distribution is restricted to the cold desert regions of northern Xinjiang [22]. The species exhibits pronounced diaspore polymorphism, with the dispersal unit consisting of a seed enclosed within a persistent pericarp (anthocarp) [22]. This indehiscent, thick-walled, often winged fruit provides mechanical protection. It contributes to seed persistence and viability under harsh desert conditions, thereby playing a key role in the ecological success of the species [23,24].

*A. desertorum* is a small annual Brassicaceae species. It bears tiny, short-lived, weakly dormant seeds that are more susceptible to deterioration, making it helpful in detecting early loss of vigor [25,26]. *A. desertorum* generally shows more synchronous germination with minimal heterochrony [27,28].

The selected species differ in seed dormancy and germination strategies, with *S. affinis* showing natural germination heterochrony, whereas *C. arenarius* and *T. arcuata* exhibit moderate dormancy-driven delays, and *A. desertorum* germinates more synchronously. These differences provide a useful framework for evaluating species-specific responses to seed aging.

Natural aging provides realistic insights into seed storability but is slow and labor-intensive. In contrast, artificial aging offers a rapid and efficient method to simulate the natural deterioration process [5]. Several studies have reported that the physiological responses to artificial and natural aging are often comparable, as shown in rice and cotton seeds [29,30]. However, other studies suggest that naturally aged seeds can exhibit more pronounced physiological changes than artificially aged ones, such as in *Phaseolus vulgaris*, where protein degradation differed significantly between the two treatments [31]. These findings indicate that artificial aging may not always fully replicate the natural aging process and must be validated for each species.

Seed priming, or osmotic conditioning, is a pre-sowing treatment that partially hydrates the seeds to activate metabolism without radicle emergence, followed by re-drying. This process enhances germination speed, uniformity, and stress tolerance by inducing “priming memory.” Among various priming agents, gibberellins (GA_3_) are widely used plant growth regulators known to alleviate the negative effects of seed aging [8]. GA_3_ promotes the activation of hydrolytic enzymes in the endosperm, regulates antioxidant defense, adjusts hormonal balance, and stimulates cell division and elongation. Furthermore, the seed priming with GA could partially ameliorate the negative effects of aging on germination and growth indices of aged seeds [10,11]. Although GA_3_-based priming has been studied in several crops and horticultural species, its role in short-lived desert forage plants remains poorly understood. Key questions include: How do aged seeds respond to exogenous GA_3_? How do endogenous hormones (IAA, CTK, ABA) interact with GA_3_ during this process? Does GA_3_ act differently under natural and artificial aging conditions? And what concentration is most effective for restoring seed viability?

This study, therefore, aimed to investigate the regulatory role of gibberellin (GA_3_) in maintaining or restoring the seed viability of short-lived desert forage species under both natural and artificial aging. The specific objectives were to (1) compare the effects of natural and artificial aging on seed vigor, enzymatic activity, and hormonal balance, and (2) determine the optimal GA_3_ concentration for improving the germination and physiological performance of aged seeds. The findings will provide a scientific basis for improving seed storage, extending viability, and supporting vegetation restoration in desert ecosystems.

## 2. Results

### 2.1. Germination Indices

ANOVA revealed significant effects of species, aging, and GA_3_ concentration on germination potential (GP), germination rate (GR), germination index (GI), and vitality index (VI) (Table 1 and Table 2). Significant S × A × GA interactions were detected for GR, GI, and VI, indicating species-specific variation in sensitivity to aging and GA_3_. Aging markedly reduced all germination indices across all species. Fresh seeds (FS) consistently exhibited the highest GP, GR, GI, and VI, while naturally aged (NA) and artificially aged (AA) seeds showed progressive losses.

**Figure 1:**
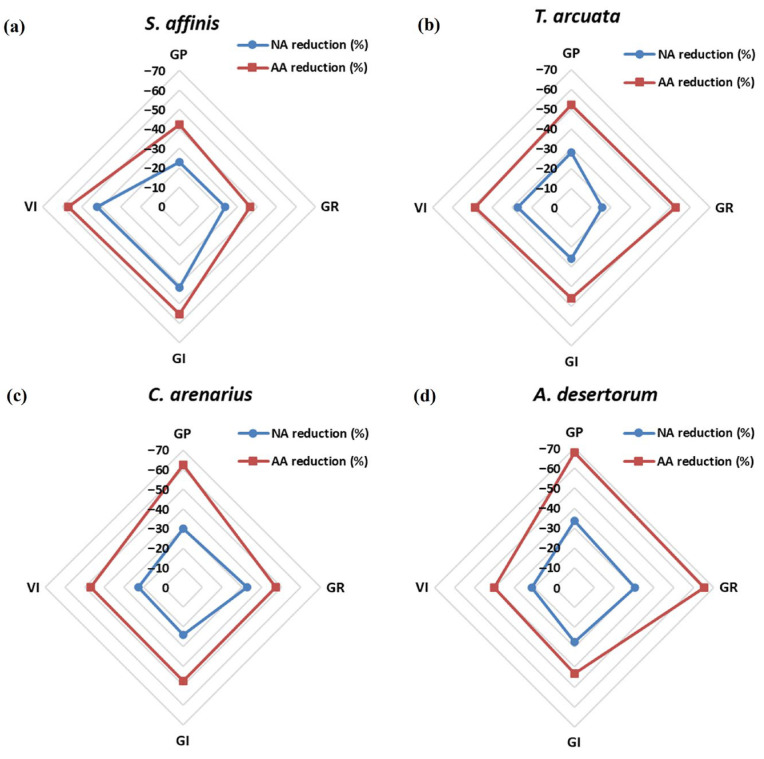
Radar plots illustrating the aging effect on germination traits, germination potential (GP), germination rate (GR), germination index (GI), and vitality index (VI), of four halophytic species: (**a**) *Salsola affinis*, (**b**) *Trigonella arcuata*, (**c**) *Ceratocarpus arenarius*, and (**d**) *Alyssum desertorum* under naturally aged (NA) and artificially aged (AA) seed conditions relative to fresh seeds (FS) under GA_0_ treatment. Values represent the percentage decrease compared with FS, with negative values indicating the magnitude of aging-induced deterioration.

Without GA, NA caused a 22.8, 28, 29.8, and 33.6% reduction in GP in *S. affinis*, *T. arcuata*, *C. arenarius*, and *A. desertorum*, respectively, as compared to FS (Figure 1; Table 1). While AA caused 42.4, 52, 62.4, and 67.8% reduction in GP in the above species. GA application showed improvement in germination indices by increasing GA up to 200 mg L^−1^ (GA200), the most stimulatory concentration. GA200 increased the GP by 22.8% in *S. affinis*, 31.7% in *T. arcuata*, 28% in *C. arenarius*, and 32% in *A. desertorum*. A slight decline or plateau occurred at GA500, particularly in *S. affinis* and *C. arenarius*. GR followed similar patterns to GP, decreasing sharply with age and increasing with GA_3_. NA reduced GR by 23.4%, 15.7%, 32.5%, and 30.4%, and AA reduced GR by 36.4, 52.8, 47.3%, and 65.2% in *S. affinis*, *T. arcuata*, *C. arenarius*, and *A. desertorum*, respectively, as compared to FS. GA200 maximized GR in all species with 14, 16, 19, and 17% increase in FS as compared to no GA.

GI integrates both speed and uniformity of germination (Table 2). NA declined the GI by 41.6, 26, 24, and 27% while AA showed 55, 46, 47.5, and 43% reductions in *S. affinis*, *T. arcuata*, *C. arenarius*, and *A. desertorum*, respectively, as compared to FS (Figure 1). GA200 again produced maximum stimulation in GI, by 11, 13, 14.4, and 18% in FSs of *S. affinis*, *T. arcuata*, *C. arenarius*, and *A. desertorum*, respectively, as compared to no GA. The stimulatory effect of GA_3_ was weaker in AA seeds but remained significant. VI reflects both germination dynamics and early seedling biomass. Under GA0, VI was reduced by 42, 27, 22.5, and 21% in NA seeds and by 57, 48.5, 47, and 40% in AA seeds of *S. affinis*, *T. arcuata*, *C. arenarius*, and *A. desertorum*, respectively, as compared to no FS (Figure 1; Table 2). VI peaks at GA200 in all species, with 17, 21.6, 22.5, and 28.5% increase in VI as compared to no GA. Despite aging, NA and AA seeds still responded positively to GA, though the magnitude was reduced by 40–60%.

### 2.2. Antioxidant Enzymes and Lipid Peroxidation

**Figure 2:**
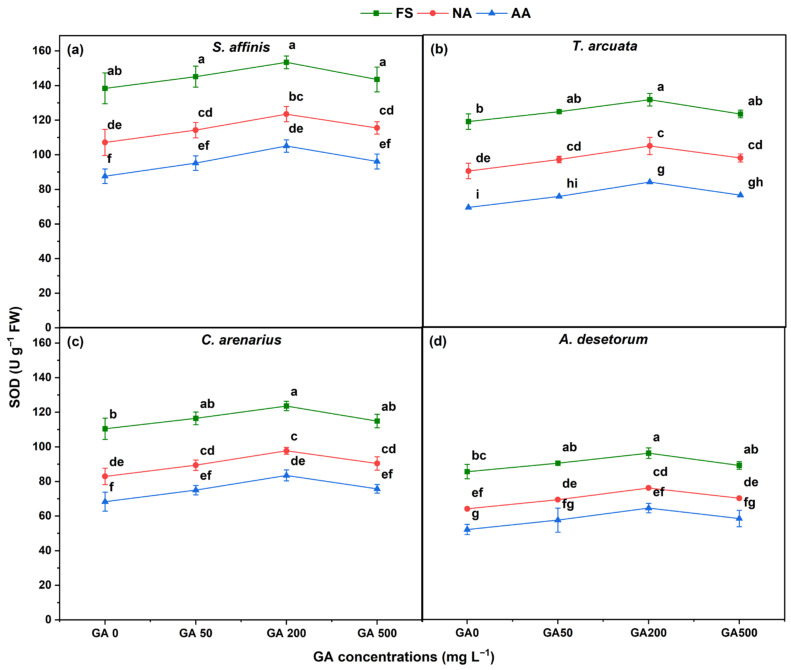
Superoxide dismutase (SOD) activity in four halophytic species (**a**) *Salsola affinis*, (**b**) *Trigonella arcuata*, (**c**) *Ceratocarpus arenarius*, and (**d**) *Alyssum desertorum* as affected by different GA_3_ concentrations (0, 50, 200, and 500 mg L^−1^) under fresh (FS), naturally aged (NA), and artificially aged (AA) seed conditions. Values represent mean ± SE (*n* = 3). Different superscript letters denote significant differences among GA treatments and aging type within a single species according to the Tukey HSD test at *p* < 0.05.

Across all species, SOD activity responded significantly to aging and GA_3_ treatments (Figure 2). FS consistently showed the highest SOD activity. In contrast, NA and AA seeds exhibited progressively lower values, indicating a decline in intrinsic antioxidative capacity as seeds deteriorated. Overall, AA seeds consistently exhibited the lowest SOD activity among all species. SOD decreased at NA by approximately 15–20% and at AA by 30–40% in *S. affinis*, *T. arcuata*, and *C. arenarius* as compared to FS. *A. desertorum* showed the most significant decline, with NA reducing SOD by about 20% and AA by nearly 45%, reflecting its greater sensitivity to aging. GA_3_ increased SOD activity. SOD peaked under GA200 in FS, NA, and AA seeds across all four species. Relative to GA0, GA200 increased SOD activity by approximately 10–14% in FS, 7–10% under NA, and 12–15% under AA in all four species.

**Figure 3:**
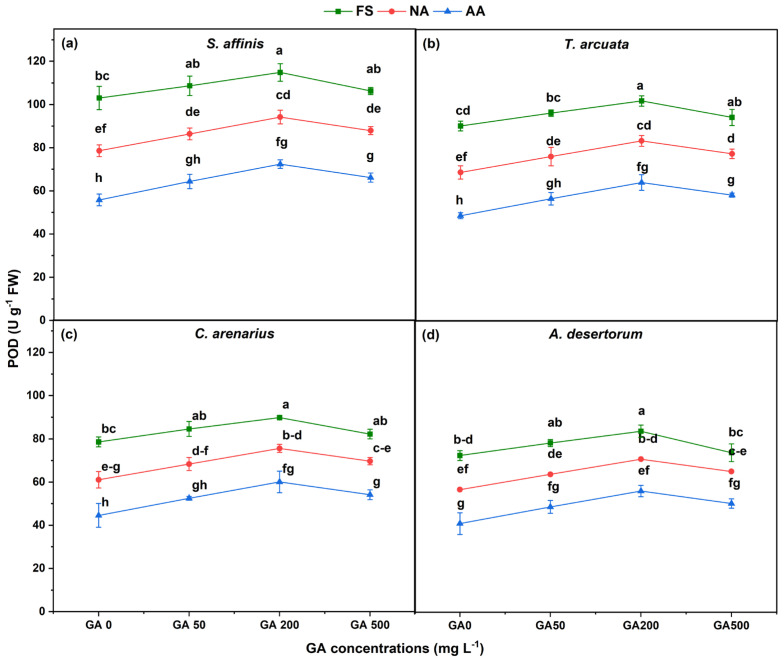
Peroxidase (POD) activity in four halophytic species (**a**) *Salsola affinis*, (**b**) *Trigonella arcuata*, (**c**) *Ceratocarpus arenarius*, and (**d**) *Alyssum desertorum* as affected by different GA_3_ concentrations (0, 50, 200, and 500 mg L^−1^) under fresh (FS), naturally aged (NA), and artificially aged (AA) seed conditions. Values represent mean ± SE (*n* = 3). Different superscript letters denote significant differences among GA treatments and aging type within a single species according to the Tukey HSD test at *p* < 0.05.

POD activity exhibited trends similar to SOD, with FS > NA > AA across all GA concentrations (Figure 3). NA seeds showed a 12–18% decrease in POD while AA seeds exhibited a 25–35% decrease, relative to FS, depending on species. The largest reduction was observed in *A. desertorum*, where POD declined by 35% in AA seeds. *S. affinis* and *T. arcuata* showed slightly smaller but still substantial AA-mediated reductions. POD activity increased under GA_3_, with GA200 producing the highest values in all species. GA200 application increased POD activity by 10–15% in FSs, 20–24.8% in NA, and 29.7–36.7% increase in AA seeds across all species.

**Figure 4:**
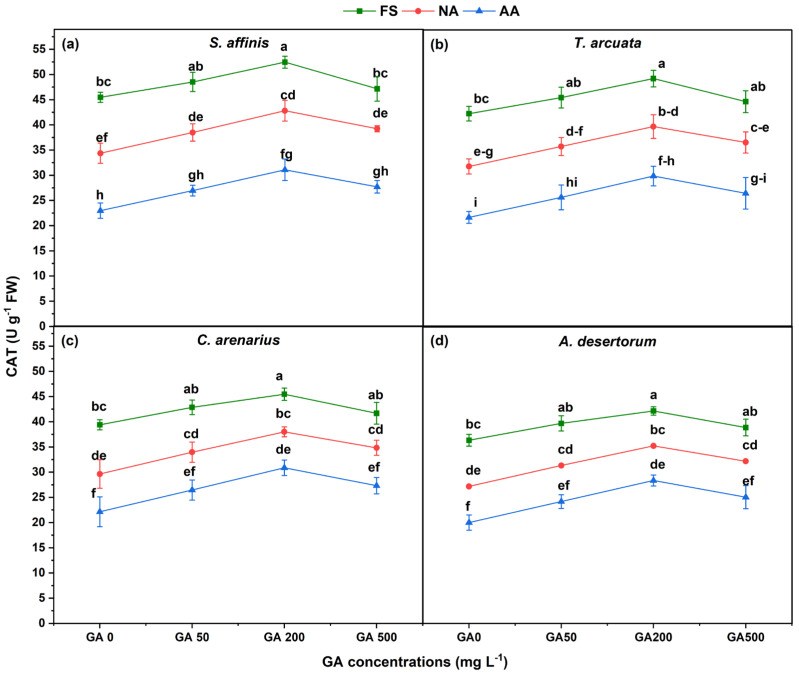
Catalase (CAT) activity in four halophytic species (**a**) *Salsola affinis*, (**b**) *Trigonella arcuata*, (**c**) *Ceratocarpus arenarius*, and (**d**) *Alyssum desertorum* as affected by different GA_3_ concentrations (0, 50, 200, and 500 mg L^−1^) under fresh (FS), naturally aged (NA), and artificially aged (AA) seed conditions. Values represent mean ± SE (*n* = 3). Different superscript letters denote significant differences among GA treatments and aging type within a single species according to the Tukey HSD test at *p* < 0.05.

CAT showed consistent reductions with aging, whereas GA_3_, especially GA200, improved activity (Figure 4). At GA0, NA reduced CAT by 15–20%, and AA reduced it by 30–40% in all species. *S. affinis* retained the highest CAT levels even under artificial aging, whereas *A. desertorum* again showed the largest decline. GA200 increased CAT to its maximum across all species and aging levels. GA200 increased CAT by 15.3, 16.4, 15.4, and 16% in FSs, 24.6, 24.8, 28.2, 29.6% in NA seeds, and 35.2, 37.8, 39.5, 41.7% increase in AA seeds, as compared to GA0.

**Figure 5:**
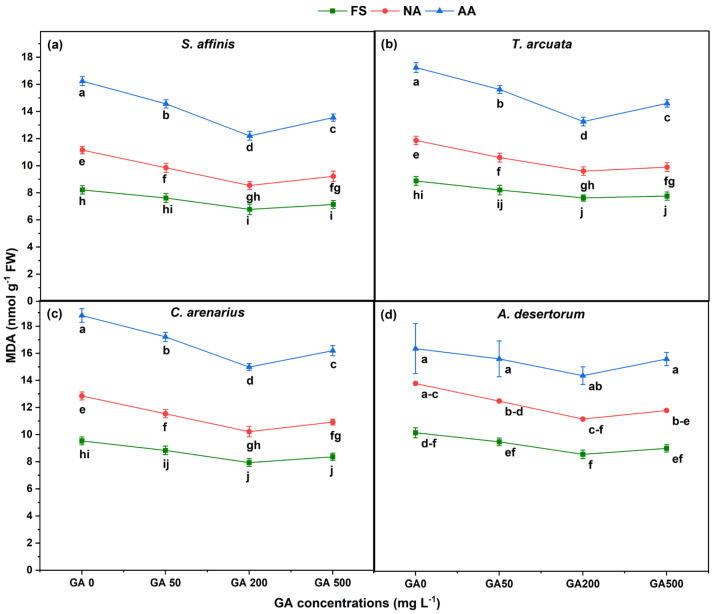
Malondialdehyde (MDA) content in four halophytic species (**a**) *Salsola affinis*, (**b**) *Trigonella arcuata*, (**c**) *Ceratocarpus arenarius*, and (**d**) *Alyssum desertorum* as affected by different GA_3_ concentrations (0, 50, 200, and 500 mg L^−1^) under fresh (FS), naturally aged (NA), and artificially aged (AA) seed conditions. Values represent mean ± SE (*n* = 3). Different superscript letters denote significant differences among GA treatments and aging type within a single species according to the Tukey HSD test at *p* < 0.05.

MDA, a lipid peroxidation marker, showed strong and inverse trends compared with antioxidant enzymes (Figure 5). MDA content increased with aging following the pattern FS < NA < AA. MDA increased by 15–20% in NA seeds and 35–45% in AA seeds as compared to FSs in *S. affinis*, *T. arcuata*, and *C. arenarius*. *A. desertorum* showed the most pronounced rise (~50%), confirming its higher oxidative vulnerability during deterioration.

GA treatments, especially GA200, significantly reduced MDA relative to GA0 across all species and aging levels. Under GA200, FSs showed 8–12% reductions in MDA, NA seeds showed 10–15% reductions, and AA seeds exhibited 12–18% reductions, though values remained higher than those of FS.

### 2.3. Hormone Content

**Figure 6:**
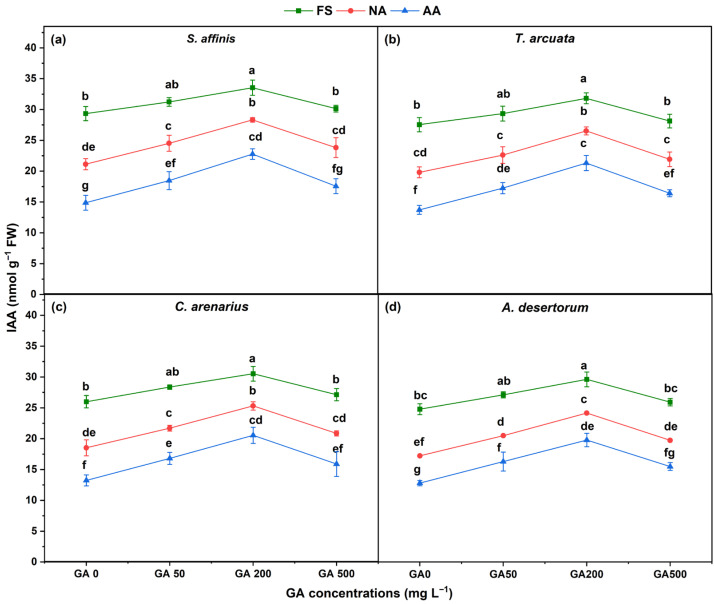
Indole-3-acetic acid (IAA) in four halophytic species (**a**) *Salsola affinis*, (**b**) *Trigonella arcuata*, (**c**) *Ceratocarpus arenarius*, and (**d**) *Alyssum desertorum* as affected by different GA_3_ concentrations (0, 50, 200, and 500 mg L^−1^) under fresh (FS), naturally aged (NA), and artificially aged (AA) seed conditions. Values represent mean ± SE (*n* = 3). Different superscript letters denote significant differences among GA treatments and aging type within a single species according to the Tukey HSD test at *p* < 0.05.

Indole-3-acetic acid (IAA) content was significantly affected by seed aging, GA_3_ concentration, and their interaction across all four species (Figure 6). FS consistently exhibited the highest IAA levels, whereas NA and AA seeds showed marked reductions, indicating progressive impairment of auxin homeostasis with aging. NA and AA declined IAA by 28 and 49.3% in *S. affinis*, 28.1 and 50.2% in *T. arcuata*, 28.7% and 49.1% in *C. arenarius*, and 30.6% and 48.4% in *A. desertorum* as compared to FS. IAA increased significantly with GA_3_ up to GA200, followed by a decline at GA500. At GA200, IAA increased in FS by 14.3, 15.5, 17.5, and 19.5% in *S. affinis*, *T. arcuata*, *C. arenarius*, and *A. desertorum*, respectively, as compared to FS. Similarly, in NA seeds, GA200 enhanced IAA by 34.1%, 33.9%, 36.6%, and 40.3%, respectively. In AA seeds, the increase reached 53.0%, 55.4%, 55.2%, and 54.7%, indicating stronger GA responsiveness under advanced aging. Overall, *S. affinis* maintained the highest IAA levels, whereas *A. desertorum* showed the greatest proportional recovery under GA_3_ despite lower baseline values.

**Figure 7:**
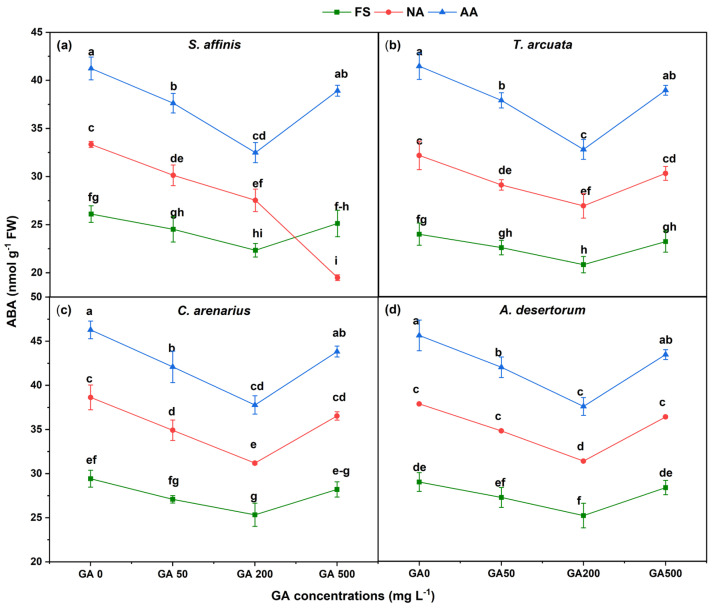
Abscisic acid (ABA) in four halophytic species (**a**) *Salsola affinis*, (**b**) *Trigonella arcuata*, (**c**) *Ceratocarpus arenarius*, and (**d**) *Alyssum desertorum* as affected by different GA_3_ concentrations (0, 50, 200, and 500 mg L^−1^) under fresh (FS), naturally aged (NA), and artificially aged (AA) seed conditions. Values represent mean ± SE (*n* = 3). Different superscript letters denote significant differences among GA treatments and aging type within a single species according to the Tukey HSD test at *p* < 0.05.

Abscisic acid (ABA) content exhibited trends opposite to IAA, increasing significantly with aging and declining in response to GA_3_ application (Figure 7). Compared with FS, ABA increased sharply in NA and AA seeds. NA seeds showed 27.7, 36.3, 31.3, and 35.2% increase in ABA in *S. affinis*, *T. arcuata*, *C. arenarius*, and *A. desertorum*, respectively, as compared to FS. Similarly, AA seeds showed 58, 77.4, 57.3, and 66.1% increase in the above species. GA_3_ significantly suppressed ABA levels, with GA200 producing the greatest reduction. In FS, ABA declined by 14.4–17.4% at GA200 relative to GA0 across species. In NA seeds, ABA decreased by 17.4, 17, 19.3, and 19.1% in *S. affinis*, *T. arcuata*, *C. arenarius*, and *A. desertorum*, respectively, as compared to GA0. In AA seeds, GA200 reduced ABA by 21.2%, 21.6%, 18.4%, and 19.3%, respectively.

**Figure 8:**
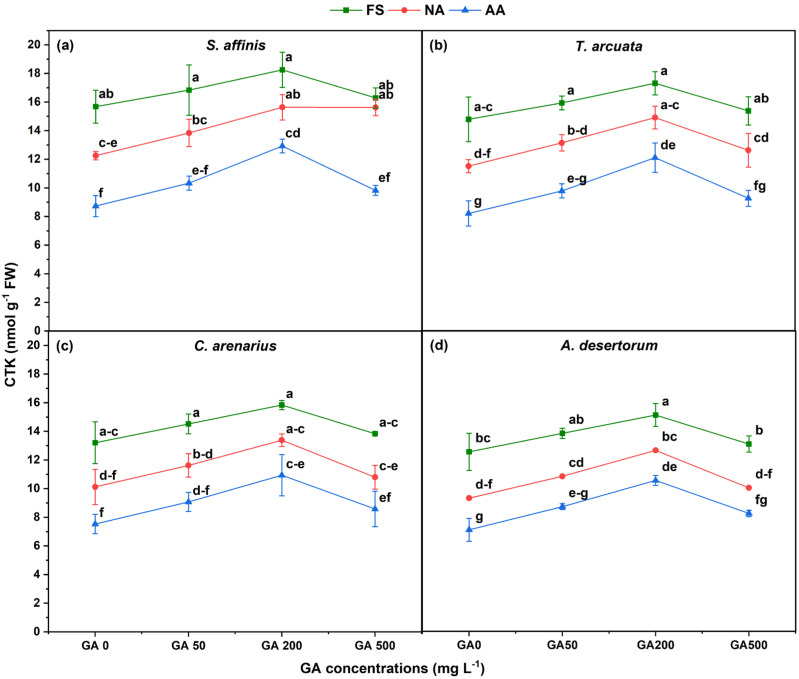
Cytokinin (CTK) in four halophytic species (**a**) *Salsola affinis*, (**b**) *Trigonella arcuata*, (**c**) *Ceratocarpus arenarius*, and (**d**) *Alyssum desertorum* as affected by different GA_3_ concentrations (0, 50, 200, and 500 mg L^−1^) under fresh (FS), naturally aged (NA), and artificially aged (AA) seed conditions. Values represent mean ± SE (*n* = 3). Different superscript letters denote significant differences among GA treatments and aging type within a single species according to the Tukey HSD test at *p* < 0.05.

Cytokinin (CTK) content declined significantly with aging but increased in response to GA_3_, particularly at GA200 (Figure 8). Relative to FS, CTK decreased by 21.8, 22, 23.4, and 25.8% in *S. affinis*, *T. arcuata*, *C. arenarius*, and *A. desertorum*, respectively, in NA seed, and by 44.3, 44.5, 43, and 43.5% in AA seeds, as compared to FSs. These reductions highlight strong age-dependent suppression of cytokinin biosynthesis or stability. GA_3_ application significantly enhanced CTK, with GA200 yielding maximal values. In FSs, CTK increased by 16.4, 17, 20, and 20.5% % in *S. affinis*, *T. arcuata*, *C. arenarius*, and *A. desertorum*, respectively, relative to GA0. In NA seeds, GA200 increased CTK by 27.6–35.9%, while in AA seeds, increases reached 48.1–60.6%, demonstrating strong GA-mediated hormonal recovery under aging stress.

### 2.4. Multivariate Analysis

#### 2.4.1. Principal Component Analysis (PCA)

Principal component analysis was performed to summarize the relationships among germination traits, antioxidant enzymes, lipid peroxidation, and phytohormones under different aging and GA_3_ treatments. The PCA results showed a strong dimensional reduction, with the first two principal components explaining 96.37% of the total variance (Table 3). PC1 had an eigenvalue of 10.20 and accounted for 92.69% of the total variance, indicating that most of the variation among treatments was driven by a common response pattern. PC2 explained an additional 3.69% of the variance, while all remaining components individually contributed less than 2% and were therefore considered minor.

Loadings from Table 4 revealed that PC1 was positively associated with germination potential (GP), germination rate (GR), germination index (GI), vitality index (VI), antioxidant enzymes (SOD, POD, CAT), and growth-promoting hormones (IAA and CTK). In contrast, PC1 showed strong negative loadings for MDA and ABA, indicating that these traits varied in opposition to germination performance and antioxidant capacity.

**Figure 9:**
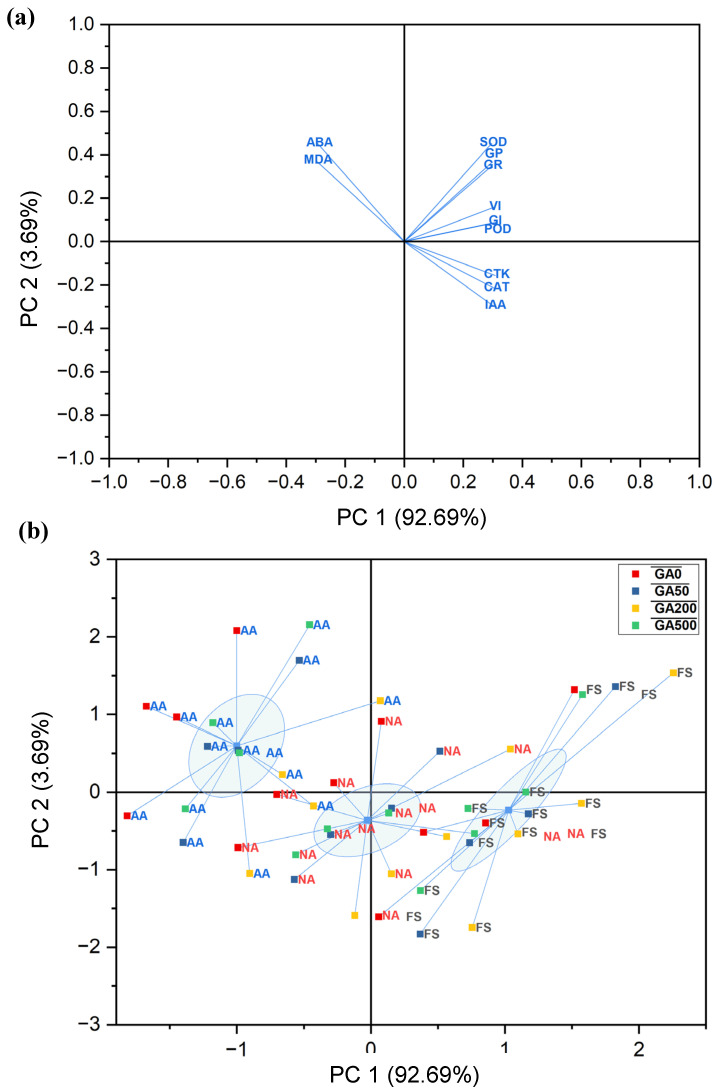
(**a**) Principal component analysis (PCA) loading plot showing the contribution of germination traits (GP, GR, GI, VI), antioxidant enzymes (SOD, POD, CAT), lipid peroxidation (MDA), and phytohormones (IAA, ABA, CTK) to the first two principal components. (**b**) PCA score plot illustrating the distribution of seed aging treatments, fresh (FS), naturally aged (NA), and artificially aged (AA), across GA_3_ concentrations (0, 50, 200, and 500 mg L^−1^).

PC2 was mainly influenced by SOD, GP, GR, ABA, and MDA, all showing positive loadings (Figure 9a). In contrast, IAA, CTK, and CAT contributed negatively, suggesting that PC2 captured secondary variation related to oxidative stress and hormonal balance rather than overall vigor.

The PCA biplot clearly separated samples according to aging status. Fresh seeds (FS) clustered on the positive side of PC1 and were closely associated with high germination traits, antioxidant activities, IAA, and CTK (Figure 9b). Naturally aged (NA) seeds occupied intermediate positions, while artificially aged (AA) seeds were located on the negative side of PC1, aligning with higher ABA and MDA levels. This distribution confirms that seed aging was the dominant factor shaping multivariate trait responses, with GA_3_ treatments shifting samples toward the positive PC1 axis.

#### 2.4.2. Correlation Analysis

**Figure 10:**
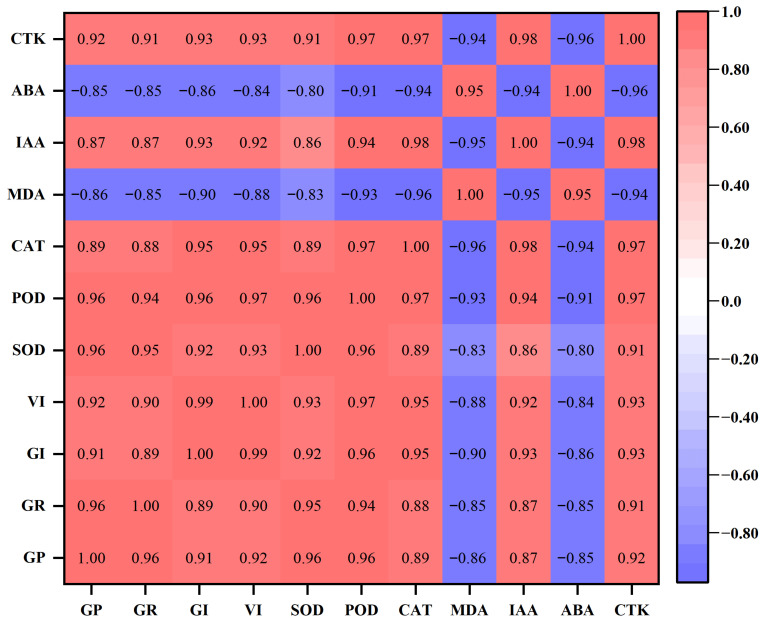
Pearson correlation matrix showing relationships among germination traits (GP, GR, GI, VI), antioxidant enzymes (SOD, POD, CAT), lipid peroxidation (MDA), and phytohormones (IAA, ABA, CTK). Positive correlations are represented in red and negative correlations in blue, with the color intensity indicating the strength of the correlation (−1 to +1).

Correlation analysis further supported the PCA results by revealing strong and consistent relationships among traits (Figure 10). All germination parameters (GP, GR, GI, and VI) were strongly and positively correlated with antioxidant enzymes (SOD, POD, and CAT), with correlation coefficients generally exceeding 0.88. These traits also showed strong positive correlations with IAA and CTK, indicating close coordination between hormonal regulation, antioxidant defense, and germination performance.

In contrast, MDA exhibited strong negative correlations with GP, GR, GI, VI, and antioxidant enzymes (r ≈ −0.83 to −0.96), reflecting the inhibitory role of lipid peroxidation on seed vigor. Similarly, ABA showed strong negative correlations with germination traits, antioxidant enzymes, IAA, and CTK, but a positive correlation with MDA, highlighting its association with stress accumulation and aging-related deterioration. Notably, IAA and CTK were highly positively correlated with each other and with antioxidant enzymes, whereas both hormones were strongly negatively correlated with ABA and MDA. These relationships indicate a clear hormonal–physiological axis in which growth-promoting hormones and antioxidant capacity jointly support germination, while oxidative damage and ABA accumulation are linked to reduced performance.

## 3. Discussion

### 3.1. Seed Aging Imposes a Progressive Decline in Germination Performance Across Species

The results clearly demonstrate that seed aging exerted a strong and consistent adverse effect on all germination indices across the four desert species. Fresh seeds (FS) consistently exhibited the highest germination potential (GP), germination rate (GR), germination index (GI), and vitality index (VI). In contrast, naturally aged (NA) and artificially aged (AA) seeds showed a stepwise decline in seed quality. NA seeds showed a moderate but significant reduction, whereas AA seeds exhibited a much more severe decline. Under GA_0_ conditions, NA seeds showed 22–34% reductions in germination indices, whereas artificial aging caused severe losses ranging from 40% to over 65%, with *A. desertorum* being the most aging-sensitive species. The decline from NA to AA confirms that artificial aging accelerated viability loss beyond natural aging.

The coordinated decline in GP, GR, GI, and VI suggests that aging disrupted multiple physiological processes simultaneously rather than a single germination-related function [5,8]. This interpretation is supported by Soltani et al. [32], who reported that seed aging affected both emergence dynamics and subsequent seedling growth, indicating that deterioration extends beyond loss of viability to include delayed establishment and reduced growth potential. This coordinated decline across multiple germination indices is consistent with the contemporary view that seed aging is a progressive, system-level process involving integrated deterioration of metabolic coordination, hormonal regulation, and cellular repair capacity rather than isolated impairment of individual traits [11].

Pirredda et al. [11] emphasized that reductions in germination speed, uniformity, and vigor reflect cumulative disruptions in reserve mobilization, membrane integrity, redox homeostasis, and hormonal signaling that intensify with aging duration and severity. Consistent with this multi-process disruption, da Silva et al. [33] demonstrated that aging-induced loss of seed vigor is tightly linked to changes in seed biochemical composition, particularly reductions in soluble proteins and increased membrane instability, both of which constrain early seedling development.

At the metabolic level, aging-related impairment of reserve mobilization likely contributed to the observed reductions in germination indices. Padilha et al. [34] demonstrated that artificial aging significantly reduced α-amylase activity, thereby limiting starch hydrolysis and energy supply to the embryonic axis during early germination. When considered together, reduced reserve mobilization [34] and altered biochemical composition under aging [33] provide a mechanistic basis for the strong reductions in GR, GI, and VI observed in the present study.

Artificial aging caused more severe reductions in germination indices than natural aging across all species, reflecting the accelerated nature of deterioration [5]. This distinction aligns with the aging continuum proposed by Pirredda et al. [11], who described artificial aging as an accelerated deterioration state that rapidly overwhelms intrinsic repair and buffering mechanisms. In contrast, natural aging proceeds more gradually and may allow for partial physiological compensation. Accordingly, artificial aging disproportionately amplifies oxidative damage, hormonal imbalance, and membrane destabilization, resulting in sharper declines in germination initiation and speed than those observed under natural aging.

The higher MDA accumulation and stronger ABA dominance observed under artificial aging indicate extensive membrane and metabolic disruption, which likely exceeds the seed’s intrinsic capacity for physiological adjustment. In line with this, da Silva et al. [33] showed that artificial aging triggers deterioration through distinct and more aggressive biochemical pathways than natural aging, resulting in faster loss of vigor even when the final germination percentage appears less affected. Consequently, artificial aging represents an intensified deterioration scenario, resulting in more pronounced impairment of germination initiation, speed, and seedling vigor.

This interpretation is consistent with ecological and physiological evidence that delayed or impaired germination in aged seeds reflects the time required for internal repair of damaged membranes and metabolic systems prior to radicle emergence [3,35]. The stronger reductions observed under artificial aging compared with natural aging are therefore consistent with previous reports showing that accelerated aging intensifies deterioration of both germination capacity and emergence rate [32].

Trait sensitivity to aging differed among species. In *S. affinis*, aging predominantly caused stronger reductions in GI and VI. This suggests that although some seeds retained the ability to germinate, aging strongly disrupted metabolic efficiency and post-germination performance, likely due to reduced antioxidant repair capacity, elevated lipid peroxidation, and limited energy availability. Whereas in *A. desertorum*, GP and GR were most severely reduced, suggesting that aging predominantly affected germination initiation mechanisms, such as hormonal signaling and early membrane repair, rather than post-germination growth capacity. Once germination occurred, seedlings were comparatively better able to maintain biomass accumulation, resulting in relatively smaller declines in GI and VI. These species-specific aging responses are summarized in the radar plots (Figure 1). Consistent with the seed aging/repair hypothesis proposed by Powell and Matthews [35], reductions in vigor-related traits were more pronounced in some species than reductions in final germination capacity, highlighting differential sensitivity of germination processes during aging.

### 3.2. GA_3_ Application Partially Restores Germination Capacity, with GA200 as the Optimal Concentration

Exogenous GA_3_ significantly alleviated aging-induced losses in germination indices across all species and aging levels. The concentration-dependent response to GA_3_ observed in the present study is consistent with evidence that aging suppresses endogenous GA biosynthesis and signaling, thereby limiting germination recovery unless an optimal external dose is supplied. Kamaei and Kafi [36] demonstrated that accelerated aging significantly reduced endogenous GA_1_ levels while downregulating GA biosynthesis and signaling genes (e.g., GA1, GA3ox), resulting in delayed and reduced germination.

Within the broader framework of seed longevity regulation, Pirredda et al. [11] highlighted gibberellins as central positive regulators of post-aging germination competence through their roles in reactivating reserve mobilization, embryo growth, and antioxidant-associated repair pathways. This conceptual model supports the present observation that exogenous GA_3_ application partially compensates for aging-induced suppression of endogenous GA signaling, thereby restoring germination performance when deterioration has not exceeded repairable thresholds. The increase in soluble sugars and protein content observed in GA_3_-primed aged seeds further indicates enhanced reserve mobilization and metabolic reactivation during germination. Such biochemical improvements likely underpin the superior germination performance and vigor recovery reported following GA_3_ priming [8].

Exogenous GA_3_ application partially restored germination by reactivating GA-dependent metabolic and hormonal pathways, but excessive GA levels failed to produce further benefits once structural and oxidative damage exceeded repair capacity. This mechanistic framework explains why GA200 was optimal in the present study, whereas GA500 produced diminishing or negative returns, particularly in more deteriorated seeds. Germination recovery depends on restoring the balance between GA and ABA rather than simply increasing GA availability [37]. Moderate GA supplementation can effectively counteract ABA-mediated repression of germination by reactivating GA-responsive pathways involved in reserve mobilization and radicle elongation. In contrast, supra-optimal GA levels may trigger feedback regulation or disrupt hormonal homeostasis, explaining the plateau or slight decline in germination indices observed at GA500 [8]. The stimulatory effect increased with GA_3_ concentration up to 200 mg L^−1^, after which a plateau or slight decline was observed at GA500, particularly in *S. affinis* and *C. arenarius*. GA200 consistently produced the maximum recovery in GP, GR, GI, and VI, establishing it as the most effective concentration across species.

Notably, the relative responsiveness to GA_3_ increased with aging severity. While FSs showed moderate improvements, NA and especially AA seeds exhibited stronger proportional gains, despite not fully reaching FS levels. This pattern indicates that GA_3_ primarily mitigated aging-associated constraints rather than enhancing already optimal germination [8]. However, the reduced magnitude of response in AA seeds compared with FS confirms that advanced deterioration limits the extent of hormonal recovery. Similar to our observations, Rouhi and Sepehri [8] demonstrated that GA_3_ priming markedly restored germination and vigor in accelerated-aged groundnut seeds, with the strongest recovery observed at moderate GA_3_ concentrations. Malviya and Gayen [37] further noted that although aged seeds often show heightened responsiveness to exogenous GA due to depleted endogenous GA pools, advanced deterioration constrains hormone perception and downstream signaling, which likely explains why AA seeds in the present study did not fully recover to FS levels despite GA_3_ application. Rouhi and Sepehri [8] further reported that the coordinated antioxidant upregulation by GA_3_ was closely associated with improved germination and seedling vigor during post-stress recovery, reinforcing the role of GA_3_ in mitigating aging-induced oxidative damage

### 3.3. Oxidative Imbalance and Membrane Deterioration Jointly Regulate Seed Vigor Loss

Seed aging is accompanied by a gradual accumulation of reactive oxygen species (ROS) as metabolic regulation deteriorates during storage, leading to oxidative damage to membranes, proteins, and nucleic acids [8,37]. To limit this damage, seeds rely on their antioxidant defense system, which remains functionally important during storage and is rapidly activated during imbibition and early germination to facilitate cellular repair and metabolic reactivation [7]. Therefore, the activity of antioxidant enzymes during germination reflects the seed’s capacity to counteract aging-induced oxidative stress and successfully resume growth.

In the present study, reductions in germination performance closely paralleled declines in antioxidant enzyme activities, with SOD, POD, and CAT following a consistent FS > NA > AA pattern across species. This indicates that aging progressively weakened the antioxidative repair capacity available during germination [8]. In contrast, MDA content increased with aging, indicating intensifying lipid peroxidation and membrane deterioration that likely constrained metabolic recovery and radicle emergence. Xu et al. [9] reported a similar pattern under accelerated aging, showing that reductions in antioxidant enzyme activities were strongly and negatively correlated with MDA accumulation and germination performance, supporting the view that impaired antioxidant-mediated repair is a central driver of vigor loss rather than a secondary consequence of aging.

This opposing behavior of antioxidant enzymes and MDA was strongly supported by multivariate patterns, where germination indices, antioxidant activities, IAA, and CTK clustered together and were clearly separated from MDA and ABA, indicating two antagonistic physiological states. Similar trait groupings were reported by da Silva et al. [33], who demonstrated that biochemical indicators of oxidative damage and membrane instability load negatively against vigor-related traits under both natural and artificial aging. Likewise, Xu et al. [9] reported strong positive correlations between antioxidant enzyme activities and germination traits, alongside pronounced negative correlations between MDA accumulation and seed vigor, reinforcing the central role of oxidative balance in regulating germination performance during aging.

The particularly sharp decline in CAT and POD activity suggests that hydrogen peroxide detoxification capacity was more severely compromised than superoxide scavenging, allowing oxidative damage to propagate into membrane lipids. Elevated MDA levels, especially under artificial aging, indicate that lipid peroxidation acts as a central constraint on metabolic recovery during germination [8]. da Silva et al. [33] likewise emphasized that membrane-associated deterioration, rather than loss of reserves alone, plays a decisive role in limiting seed performance during aging.

GA_3_ application significantly mitigated oxidative imbalance by enhancing antioxidant enzyme activities while reducing MDA accumulation, with GA200 producing the strongest effect. However, even under GA_3_ treatment, MDA levels in NA and AA seeds remained higher than in FSs, indicating partial rather than complete restoration of membrane integrity [8]. The persistent negative correlations between MDA and germination traits reinforce lipid peroxidation as a primary biochemical bottleneck that cannot be fully overcome once aging-related damage exceeds a critical threshold [9,33].

### 3.4. Hormonal Imbalance Under Aging Is Closely Linked to Germination Failure

Seed aging induced a pronounced hormonal shift characterized by reductions in growth-promoting hormones (IAA and cytokinins) and a concomitant accumulation of ABA. This hormonal imbalance was consistent across species and aging levels, with artificially aged seeds exhibiting the most severe disruption. The parallel decline of IAA and cytokinins with germination indices indicates that diminished hormonal support contributed directly to reduced germination speed, uniformity, and early seedling vigor. Similar aging-induced hormonal shifts have been reported in other crop systems, where increased ABA levels and suppression of growth-promoting hormones were closely associated with reduced seed vigor and delayed germination [5,12].

Hormonal imbalance during seed aging reflects not only shifts in hormone concentrations but also coordinated changes in hormone biosynthesis, catabolism, and signaling networks. Kamaei and Kafi [36] showed that accelerated aging simultaneously increased ABA accumulation through upregulation of NCED genes while suppressing GA biosynthesis and signaling, thereby reinforcing ABA–GA antagonism and inhibiting germination-related metabolic processes. In the present study, the strong negative association between ABA and germination indices, coupled with reduced IAA and cytokinin levels, indicates that aging imposed a multi-hormonal constraint that restricted both germination initiation and post-germination vigor.

According to Malviya and Gayen [37], seed aging disrupts hormonal homeostasis primarily through enhanced ABA biosynthesis and suppression of GA, IAA, and cytokinin signaling, leading to repression of germination-associated genes such as those involved in α-amylase production, cell wall loosening, and embryo elongation. This framework provides a mechanistic explanation for the parallel decline of IAA and cytokinins with GI and VI observed in the present study, indicating that hormonal imbalance directly compromises germination quality and seedling vigor rather than merely delaying radicle emergence.

Application of GA_3_ significantly moderated these hormonal alterations, with GA200 consistently producing the strongest recovery. The restoration of IAA and cytokinins was particularly evident in naturally and artificially aged seeds, suggesting that GA_3_ effectively reactivated hormonal pathways that had been suppressed during aging [38]. At the same time, ABA content declined markedly under GA_3_ treatment, especially at GA200, although ABA levels in aged seeds remained higher than those in fresh seeds. This partial correction of hormonal balance under GA_3_ is consistent with findings by Wang B et al. [5], who demonstrated that exogenous hormonal regulation can alleviate, but not fully reverse, aging-induced hormonal and physiological constraints on germination. Malviya and Gayen [37] highlighted that exogenous GA can partially restore hormonal balance by suppressing ABA signaling and reactivating growth-promoting hormone networks, but complete recovery is unlikely once aging-induced oxidative and membrane damage disrupts hormone sensitivity. This interpretation aligns with the partial but incomplete normalization of ABA, IAA, and cytokinin levels observed in aged seeds under GA_3_ treatment in the present study.

Kamaei and Kafi [36] demonstrated that GA treatment reduced ABA dominance and restored GA-associated gene expression, but could not fully reverse aging-induced oxidative and membrane damage. This explains why GA_3_ significantly improved germination performance in aged seeds in the present study, yet failed to restore germination indices to fresh-seed levels.

Among species, *S. affinis* maintained the highest absolute IAA levels across aging treatments, whereas *A. desertorum* exhibited the greatest proportional hormonal recovery following GA_3_ application, despite having the lowest baseline hormone levels. This distinction helps explain why species differed in absolute germination performance yet showed broadly comparable responsiveness to GA_3_, highlighting that species-specific baseline hormone status modulates aging sensitivity while exogenous GA_3_ can partially restore germination competence across diverse ecological strategies.

The present findings have practical implications for both seed technology and ecological restoration in arid environments. The identification of GA200 as the most effective concentration for improving germination of aged seeds provides a simple strategy to enhance the establishment of desert plant species used in restoration programs. In many desert restoration projects, seeds may experience storage-related deterioration before sowing, which reduces germination success. The ability of moderate GA_3_ treatment to partially restore germination performance suggests that GA_3_ priming could be used as a practical seed treatment to improve seed vigor and field establishment. Furthermore, these findings also contribute to understanding how oxidative stress, antioxidant defense, and hormonal balance interact to regulate seed aging and germination recovery in desert species.

## 4. Materials and Methods

### 4.1. Plant Materials and Seed Collection

The seeds of four short-lived desert forage species: (i) *Salsola affinis,* (ii) *Trigonella arcuata* C.A. Mey, (iii) *Ceratocarpus arenarius,* and (iv) *Alyssum desertorum* were collected from the temperate desert region of the Nanshan low-mountain zone, Shihezi City, Xinjiang (84°58–86°24′ E, 43°26′–45°20′ N; 600–900 m a.s.l.) (Table 5). This region is characterized by a temperate continental arid climate with an average annual temperature of 6.7 °C and mean annual precipitation of approximately 351 mm.

For *Salsola affinis*, which naturally produces both winged and non-winged seeds, only fully developed winged seeds were collected for experimentation to ensure uniformity and consistency among treatments. Seeds of all species were harvested in 2023, manually cleaned, and air-dried. Well-filled, healthy seeds were selected, and their moisture content was adjusted to 10–12% according to the International Rules for Seed Testing [39]. The selected seeds were divided into three groups: (1) freshly harvested seeds (FS), (2) naturally aged seeds (NA), and (3) artificially aged seeds (AA).

### 4.2. Aging Treatments

For natural aging (NA), seeds were sealed in polyethylene bags and stored under ambient desert conditions in a dark, dry, and well-ventilated seed storage room at the Pasture Training Base of Shihezi University. The seeds were aged for three months at an average temperature of 10–15 °C and relative humidity of 35–39%. For artificial aging (AA), seeds were placed in a controlled-environment aging chamber at 41 °C and 100% relative humidity for three days. This treatment accelerated the aging process and simulated long-term natural deterioration [40]. After aging, the seeds were air-dried back to their original moisture content and stored at 4 °C until further analysis.

### 4.3. Gibberellin Treatment

Both naturally and artificially aged seeds, along with fresh seeds, were subjected to gibberellin (GA_3_) priming. Before treatment, seeds were surface sterilized by immersion in absolute ethanol for 5 min, followed by three rinses with distilled water. Excess surface moisture was removed using sterile filter paper. The sterilized seeds were then soaked in GA_3_ solutions of four concentrations: 0 mg L^−1^ (distilled water, CK), 50 mg L^−1^ (GA50), 200 mg L^−1^ (GA200), and 500 mg L^−1^ (GA500). A seed-to-solution ratio of 1:5 (*w*/*v*) was maintained. The soaking was carried out at 20 °C in darkness for 12 h, with gentle agitation every 2 h to ensure uniform contact between seeds and the solution. After treatment, the seeds were rinsed three times with sterile distilled water, gently blotted dry, and air-dried at room temperature to their original weight before germination testing.

### 4.4. Germination Test and Vigor Assessment

Germination assays were conducted to evaluate the effects of aging and GA_3_ treatments on seed viability. For each species, 100 uniform seeds were placed in 9 cm Petri dishes lined with double-layer filter paper moistened with deionized water. Each treatment was replicated three times. The Petri dishes were incubated in a growth chamber maintained at 25 °C with a 16 h light:8 h dark photoperiod, 200 µmol m^−2^ s^−1^ PPFD at seed level, and a relative humidity of 70%. Water was replenished regularly by weighing to compensate for evaporation.

The following germination parameters were recorded: germination potential (GP) on day 4, germination rate (GR) on day 7, germination index (GI), and vigor index (VI) calculated according to Guan et al. [41]:

(1) $$GP=\frac{G_{4}}{N}\times 100$$

(2) $$GR=\frac{G}{N}\times 100$$

where G_4_ and G are the number of germinated seeds on day 4 and the final number of germinated seeds (day 7), whereas N is the number of seeds tested.

(3) $$GI=\sum \frac{G_{t}}{D_{t}}$$

where G_t_ represents the number of seeds germinated on day D_t_

(4) VI=GI×SW

where SW is the mean fresh weight (g) of normal seedlings after 7 days.

### 4.5. Determination of Enzymatic and Hormonal Parameters

To assess physiological changes during germination, fresh seedlings were collected from each treatment. Samples were rinsed with phosphate buffer (pH 7.0), blotted dry, and weighed. Approximately 0.5 g of seed tissue was homogenized in 4 mL of chilled phosphate buffer (1:8, *w*/*v*) using a homogenizer at 13,000 rpm under an ice bath. The homogenate was centrifuged at 3000 rpm for 10 min at 4 °C, and the supernatant was collected for biochemical assays.

The activities of superoxide dismutase (SOD), peroxidase (POD), and catalase (CAT), as well as the content of malondialdehyde (MDA), were determined using standard spectrophotometric methods, and all results were expressed as enzyme U g^−1^ FW. SOD activity was measured by monitoring the inhibition of nitro blue tetrazolium (NBT) photoreduction at 560 nm following the method of Giannopolitis and Ries [42]. One unit (U) of SOD activity was defined as the amount of enzyme required to cause 50% inhibition of NBT reduction under the assay conditions.

POD activity was assayed by recording the increase in absorbance at 470 nm due to the oxidation of guaiacol in the presence of hydrogen peroxide, as described by Chance and Maehly [43]. One unit of POD activity corresponded to an absorbance change of 0.01 min^−1^ g^−1^ FW.

CAT activity was determined by monitoring the decomposition of H_2_O_2_ at 240 nm using a UV–visible spectrophotometer (UV-1800, Shimadzu, Kyoto, Japan) with quartz cuvettes, following the method described by Aebi [44]. One unit of CAT activity was defined as the amount of enzyme causing a decrease in absorbance of 0.01 min^−1^ g^−1^ FW.

MDA content, an indicator of lipid peroxidation, was quantified using the thiobarbituric acid (TBA) reaction described by Heath and Packer [45]. The absorbance of the supernatant was measured at 532 nm and corrected for nonspecific turbidity at 600 nm, and the MDA concentration was calculated using an extinction coefficient of 155 mM^−1^ cm^−1^. Endogenous hormone concentrations, including indole-3-acetic acid (IAA), cytokinin (CTK), and abscisic acid (ABA), were quantified using commercial enzyme-linked immunosorbent assay (ELISA) kits (J4994-A, J4947-A, and J4924-A; Jingmei Biotechnology, Lishui, China) according to the manufacturer’s protocols.

### 4.6. Statistical Analysis

All experiments were conducted in triplicate. Data were expressed as mean ± standard deviation (SD). Statistical analyses were performed using one-way analysis of variance (ANOVA), and treatment means were compared using the least significant difference (LSD) test at *p* < 0.05.

## 5. Conclusions

Collectively, the results demonstrate that seed aging reduces germination capacity through a coordinated decline in antioxidant defense and growth-promoting hormones, accompanied by enhanced lipid peroxidation and ABA accumulation. GA_3_ application, particularly at 200 mg L^−1^, partially restores this balance by enhancing antioxidant enzymes, elevating IAA and CTK, suppressing ABA and MDA, and thereby improving germination dynamics and seedling vigor. The consistency of these patterns across species, aging treatments, and multivariate analyses underscores a robust physiological framework underlying seed deterioration and GA_3_-mediated recovery.

## Figures and Tables

**Table 1:** Effects of gibberellic acid (GA_3_) concentrations and seed aging on germination percentage (GP) and germination rate (GR) of four halophytic species under fresh (FS), naturally aged (NA), and artificially aged (AA) conditions.

Species	GA	GP			GR		
		FS	NA	AA	FS	NA	AA
*S. affinis*	GA0	37.23 ± 2.11 ^cd^	28.73 ± 1.27 ^fg^	21.43 ± 1.25 ^i^	58.53 ± 2.11 ^b^	44.83 ± 2.91 ^ef^	37.23 ± 1.25 ^g^
	GA50	40.37 ± 0.91 ^bc^	32.30 ± 1.35 ^ef^	24.50 ± 0.89 ^hi^	62.07 ± 1.77 ^ab^	47.63 ± 2.29 ^d–f^	43.83 ± 1.74 ^f^
	GA200	45.70 ± 1.97 ^a^	35.97 ± 1.25 ^de^	29.47 ± 0.91 ^fg^	66.67 ± 0.85 ^a^	57.27 ± 0.87 ^bc^	52.13 ± 1.35 ^cd^
	GA500	42.60 ± 0.70 ^ab^	33.43 ± 1.37 ^e^	28.27 ± 0.35 ^gh^	63.87 ± 1.83 ^a^	49.30 ± 0.92 ^de^	46.47 ± 2.35 ^ef^
*T. arcuata*	GA0	29.73 ± 1.15 ^c^	21.40 ± 1.30 ^fg^	14.27 ± 1.26 ^h^	51.33 ± 1.15 ^bc^	43.27 ± 0.50 ^ef^	24.23 ± 1.25 ^i^
	GA50	34.83 ± 0.91 ^b^	24.87 ± 1.25 ^de^	18.30 ± 0.70 ^g^	54.63 ± 0.91 ^b^	46.40 ± 1.20 ^de^	29.50 ± 1.30 ^h^
	GA200	39.17 ± 1.00 ^a^	27.27 ± 0.87 ^cd^	22.53 ± 0.93 ^ef^	59.57 ± 1.00 ^a^	49.07 ± 0.61 ^cd^	35.77 ± 1.25 ^g^
	GA500	38.40 ± 1.30 ^a^	25.20 ± 1.30 ^de^	20.57 ± 1.11 ^fg^	52.10 ± 1.80 ^bc^	40.93 ± 1.15 ^f^	22.93 ± 2.77 ^i^
*C. arenarius*	GA0	25.03 ± 1.40 ^bc^	17.57 ± 0.85 ^ef^	9.40 ± 0.89 ^h^	43.23 ± 1.12 ^b^	29.17 ± 1.46 ^de^	22.77 ± 1.25 ^f^
	GA50	28.90 ± 1.30 ^ab^	20.43 ± 1.32 ^de^	12.97 ± 1.78 ^gh^	45.50 ± 1.70 ^b^	32.50 ± 0.89 ^cd^	27.33 ± 1.46 ^ef^
	GA200	32.03 ± 0.25 ^a^	21.63 ± 1.83 ^cd^	18.93 ± 1.74 ^de^	51.47 ± 2.90 ^a^	36.10 ± 1.15 ^c^	33.50 ± 1.30 ^cd^
	GA500	28.37 ± 1.42 ^ab^	18.30 ± 1.30 ^d–f^	14.40 ± 1.45 ^fg^	51.10 ± 1.28 ^a^	31.50 ± 1.76 ^c–e^	29.10 ± 2.33 ^de^
*A. desertorum*	GA0	21.87 ± 0.00 ^bc^	14.53 ± 0.93 ^fg^	7.03 ± 1.72 ^h^	39.57 ± 1.46 ^cd^	27.53 ± 1.14 ^g^	13.77 ± 1.25 ^j^
	GA50	23.57 ± 0.00 ^b^	18.10 ± 0.89 ^de^	11.63 ± 0.93 ^g^	42.33 ± 1.37 ^bc^	31.50 ± 1.30 ^f^	18.23 ± 1.25 ^i^
	GA200	28.87 ± 0.00 ^a^	20.13 ± 1.01 ^cd^	15.70 ± 1.30 ^ef^	46.40 ± 1.90 ^a^	36.77 ± 1.25 ^de^	23.50 ± 1.30 ^h^
	GA500	27.47 ± 0.00 ^a^	18.43 ± 0.68 ^de^	12.23 ± 1.37 ^g^	44.43 ± 0.76 ^ab^	33.77 ± 1.25 ^ef^	20.27 ± 1.25 ^hi^
ANOVA	Species (S)	1071.05 ***				1249.02 ***	
(*F*-value)	Aging (A)	1852.5 ***				2453.35 ***	
	GA	229.77 ***				225.19 ***	
	S × A × GA	1.20 ^ns^				1.73 ***	

Values are mean ± SD (*n* = 3). Different superscript letters denote significant differences among treatments according to LSD at *p* < 0.05. ANOVA results show the main and interactive effects of species (S), aging (A), and GA level. ns = non-significant; *** *p* < 0.001.

**Table 2:** Influence of GA_3_ concentrations and seed aging on germination index (GI) and vitality index (VI) of four halophytic species evaluated under fresh (FS), naturally aged (NA), and artificially aged (AA) conditions.

Species	GA	GI			VI		
		FS	NA	AA	FS	NA	AA
*S. affinis*	GA0	8.51 ± 0.17 ^b^	4.97 ± 0.15 ^ef^	3.83 ± 0.25 ^h^	3.27 ± 0.07 ^b^	1.89 ± 0.06 ^fg^	1.41 ± 0.09 ^h^
	GA50	8.82 ± 0.20 ^ab^	5.50 ± 0.36 ^de^	4.23 ± 0.15 ^gh^	3.45 ± 0.08 ^b^	2.14 ± 0.14 ^ef^	1.61 ± 0.06 ^h^
	GA200	9.42 ± 0.19 ^a^	6.13 ± 0.15 ^d^	4.67 ± 0.21 ^fg^	3.82 ± 0.08 ^a^	2.49 ± 0.06 ^d^	1.89 ± 0.08 ^fg^
	GA500	7.03 ± 0.21 ^c^	5.57 ± 0.35 ^de^	4.33 ± 0.31 ^f–h^	2.81 ± 0.08 ^c^	2.19 ± 0.14 ^e^	1.67 ± 0.12 ^gh^
*T. arcuata*	GA0	6.23 ± 0.15 ^b^	4.63 ± 0.31 ^d^	3.37 ± 0.25 ^f^	2.31 ± 0.06 ^cd^	1.68 ± 0.11 ^g^	1.19 ± 0.09 ^i^
	GA50	6.53 ± 0.23 ^ab^	5.23 ± 0.12 ^c^	3.77 ± 0.21 ^ef^	2.47 ± 0.09 ^bc^	1.95 ± 0.04 ^f^	1.39 ± 0.08 ^hi^
	GA200	7.03 ± 0.21 ^a^	5.63 ± 0.15 ^c^	4.17 ± 0.12 ^de^	2.81 ± 0.08 ^a^	2.21 ± 0.06 ^de^	1.65 ± 0.05 ^g^
	GA500	6.63 ± 0.12 ^ab^	5.37 ± 0.12 ^c^	3.97 ± 0.15 ^e^	2.57 ± 0.04 ^b^	2.03 ± 0.04 ^ef^	1.49 ± 0.06 ^gh^
*C. arenarius*	GA0	5.77 ± 0.21 ^bc^	4.37 ± 0.21 ^ef^	3.03 ± 0.21 ^h^	2.09 ± 0.08 ^cd^	1.62 ± 0.08 ^f^	1.11 ± 0.08 ^h^
	GA50	6.17 ± 0.15 ^ab^	4.67 ± 0.15 ^e^	3.43 ± 0.25 ^gh^	2.29 ± 0.06 ^bc^	1.79 ± 0.06 ^ef^	1.31 ± 0.10 ^gh^
	GA200	6.60 ± 0.10 ^a^	5.27 ± 0.06 ^cd^	3.97 ± 0.15 ^fg^	2.56 ± 0.04 ^a^	2.13 ± 0.02 ^c^	1.63 ± 0.06 ^f^
	GA500	6.33 ± 0.25 ^a^	4.87 ± 0.15 ^de^	3.53 ± 0.21 ^gh^	2.41 ± 0.10 ^ab^	1.89 ± 0.06 ^de^	1.37 ± 0.08 ^g^
*A. desertorum*	GA0	5.27 ± 0.21 ^c^	3.83 ± 0.25 ^ef^	3.00 ± 0.20 ^g^	1.79 ± 0.07 ^cd^	1.41 ± 0.00 ^fg^	1.07 ± 0.07 ^i^
	GA50	5.67 ± 0.15 ^bc^	4.27 ± 0.12 ^de^	3.33 ± 0.15 ^fg^	1.99 ± 0.05 ^b^	1.63 ± 0.00 ^de^	1.20 ± 0.06 ^hi^
	GA200	6.20 ± 0.10 ^a^	4.73 ± 0.15 ^d^	3.83 ± 0.21 ^ef^	2.30 ± 0.04 ^a^	1.91 ± 0.00 ^bc^	1.54 ± 0.08 ^ef^
	GA500	5.90 ± 0.20 ^ab^	4.33 ± 0.21 ^de^	3.47 ± 0.06 ^fg^	2.10 ± 0.07 ^b^	1.67 ± 0.00 ^de^	1.29 ± 0.02 ^gh^
ANOVA	Species (S)	437.95 ***			608.90 ***		
(*F*-value)	Aging (A)	2835.59 ***			2787.61 ***		
	GA	127.42 ***			279.53 ***		
	S × A × GA	6.46 ***			6.66 ***		

Values are mean ± SD (*n* = 3). Different superscript letters denote significant differences among treatments according to LSD at *p* < 0.05. ANOVA results show the main and interactive effects of species (S), aging (A), and GA level. *** *p* < 0.001.

**Table 3:** Eigenvalues, percentage variance explained, and cumulative variance extracted from principal component analysis (PCA) of germination, physiological, and hormonal traits.

	Eigenvalue	Variance (%)	Cumulative (%)
1	10.19539	92.69	92.69
2	0.40548	3.69	96.37
3	0.18661	1.70	98.07
4	0.06092	0.55	98.62
5	0.05116	0.47	99.09
6	0.04055	0.37	99.46
7	0.02653	0.24	99.70
8	0.01408	0.13	99.82
9	0.01187	0.11	99.93
10	0.00471	0.04	99.98
11	0.00272	0.02	100.00

**Table 4:** PCA loadings of germination traits, antioxidant enzymes, lipid peroxidation (MDA), and phytohormones contributing to principal components PC1 and PC2.

Traits	Coefficients of PC1	Coefficients of PC2
GP	0.2984	0.36596
GR	0.29583	0.34742
GI	0.30312	0.0857
VI	0.30279	0.15763
SOD	0.296	0.45224
POD	0.31109	0.08911
CAT	0.30702	−0.21624
MDA	−0.29767	0.37158
IAA	0.30332	−0.29786
ABA	−0.29275	0.45036
CTK	0.30806	−0.15607

**Table 5:** Taxonomic information of the short-lived desert forage species used in this study.

Common Name	Scientific Name	Family
Purple-winged pigweed	*Salsola affinis* C.A.Mey. ex Schrenk	Chenopodiaceae
Curved-fruit huluba	*Trigonella arcuata* C.A.Mey.	Fabaceae
Horned goosefoot	*Ceratocarpus arenarius* L.	Chenopodiaceae
Desert madwort	*Alyssum desertorum* Stapf	Brassicaceae

## Data Availability

The original contributions presented in this study are included in the article. Further inquiries can be directed to the corresponding author.
